# Impact of Job Demands, Resources, Shift Patterns, and Regulatory Focus on Nurses’ Presenteeism and Work Engagement: A Cross‐Sectional Study

**DOI:** 10.1155/jonm/5589138

**Published:** 2026-04-28

**Authors:** JooHyun Lee

**Affiliations:** ^1^ Mo-Im Kim Nursing Research Institute, College of Nursing, Yonsei University, 50-1, Yonsei-ro Seodaemun-gu, Seoul, 03722, South Korea, yonsei.ac.kr

**Keywords:** motivation, presenteeism, shift work schedule, work engagement

## Abstract

This cross‐sectional study explored how job demands, resources, shift patterns, and regulatory focus influence nurses’ presenteeism and work engagement. A sample of 176 nurses across various hospital wards participated. Emotional demands, job resources, regulatory focus, presenteeism, and work engagement were measured through surveys, while physical demands were measured using smart bands during shifts. Hierarchical regression revealed that presenteeism was positively associated with physical demands and a prevention‐dominant focus. Work engagement showed a positive correlation with relationships with supervisors and a negative correlation with prevention‐dominant focus. Including shift patterns and dominant regulatory focus significantly increased the explanatory power of the models for both outcomes. The findings highlight the importance of reassessing shift patterns, implementing effective staffing strategies, and fostering managerial support to mitigate presenteeism and enhance work engagement. Addressing organizational culture and improving job resources could contribute to nurses’ well‐being and job performance.

## 1. Introduction

The Job Demands–Resources (JD‐R) model proposes that working conditions can be categorized into two broad categories that differentially predict employee well‐being and performance [1]. Job demands are physical, psychological, social, or organizational aspects of work that require sustained effort and are associated with physiological and psychological costs. Job resources are physical, psychological, social, or organizational aspects of work that are functional in achieving work goals, reducing job demands and associated costs, or stimulating personal growth and development [2]. Thereby, job demands and resources are important determinants of a worker’s workload and strain [3].

Nurses experience both physical and emotional demands during care delivery, and the workload associated with these demands influences burnout rates and turnover intentions [4]. Empirical evidence shows that approximately 75% of hospital nurses experience moderate to high burnout [5], with burnout rates contributing to annual turnover rates of around 23% in nursing [6]. Heart rate, a physiological indicator that increases with greater physical demands, strongly correlates with energy expenditure. Heart rate increases when exercise intensity or job demands are high or perceived as requiring greater effort [7, 8]. Nurses also experience high emotional demands due to exposure to conflicts of interest arising from various interpersonal processes [9].

Despite these diverse job demands, previous studies have shown that job resources such as social support from supervisors and colleagues can buffer the negative effects of job demands [10]. Abundant job resources have been shown to alleviate presenteeism that reduces organizational productivity [11], increase work engagement, and positively influence job performance [12]. Work engagement is defined as a positive, fulfilling work‐related state characterized by vigor, dedication, and absorption [13]. On the other hand, lack of job resources makes it difficult to perform and engage in the job, leading to burnout, dissatisfaction, turnover, and work‐family conflict [14].

To address the complex interplay between organizational and individual factors, the JD‐R model has been theoretically extended to incorporate personal resources alongside organizational job resources [15]. The extended JD‐R model emphasizes that personal resources, including motivation, play a crucial role in both the health impairment process and the motivational process [15]. Motivation is the cause or trigger that induces a person to do something or be inclined to do something and is variable across individuals [16].

Regulatory focus theory provides a framework for understanding different types of motivation [17]. This theory proposes that individuals pursue goals using two distinct self‐regulatory systems based on different self‐guides and divides the human self into three parts: the actual self, which is who you think you are; the ideal self, which is your wishes, hopes, and aspirations for yourself; and the ought self, which is who you should be, even if you do not want to, because of your duties and responsibilities [17]. Promotion focus reflects motivation to reduce discrepancies between the actual self and ideal self, emphasizing growth and gain attainment through eager strategies. Prevention focus reflects motivation to reduce discrepancies between the actual self and ought self, emphasizing security and loss avoidance through vigilant strategies [17].

People with a promotion focus are more concerned with the pursuit of the ideal self, hopes, aspirations, goals, and self‐reflection, experiencing positive emotions such as pleasure and satisfaction when positive outcomes are achieved, while people with a strong prevention focus experience calm and relief when anticipated negative outcomes are avoided, and negative emotions such as anxiety and impatience when they fail [18]. An individual’s dominant regulatory focus is the one that is driven by dispositions or circumstances. In other words, regulatory focus is a theory that explains motivation in terms of the direction in which we want to reduce the difference between our desired end state and our current state, and which direction our dominant regulatory focus is in leads to differences in intentions and behavior [16]. These motivational orientations may be particularly relevant in nursing, where the high‐stakes nature of patient care and emphasis on error prevention could shape nurses’ dominant regulatory focus. However, empirical evidence integrating individual motivational orientations with job demands and resources in nursing contexts remains lacking.

Within the context of the JD‐R model, shift work represents an important organizational factor that can function as both a job demand and influence how nurses experience other workplace demands and resources. Nurses, who are required to provide 24‐h nursing care to hospitalized patients, are mostly working in shifts [19]. Shift work is a nontraditional work schedule that includes unsocial hours, such as weekends, evenings, and nights [20]. These shifts disrupt sleep, impair cognition and judgement [21], and negatively affect nurses′ performance, including decreased job performance and well‐being [22].

Excessive job demands can lead to presenteeism, defined as attending work while experiencing illness or health impairment that results in reduced work performance and productivity [23]. Previous studies have shown that as many as half of nurses experience presenteeism [24]. Shift work may exacerbate presenteeism by disrupting recovery processes and depleting personal resources needed to manage job demands effectively.

Given these multiple demands and challenges facing nurses, understanding both presenteeism and work engagement is critical because they have distinct implications for patient care and organizational outcomes. Presenteeism increases safety risks and reduces productivity, while low work engagement is associated with higher turnover intentions and compromised care quality. Identifying factors that predict both outcomes is essential for comprehensive workforce interventions. Because both organizational and individual factors jointly shape nurse outcomes, it is critical to examine how these factors together influence presenteeism and work engagement.

Despite the JD‐R model research emphasizing the need to integrate both organizational and individual factors [25], there is a lack of research that comprehensively examines whether motivational differences and shift patterns predict nurses′ presenteeism and work engagement within an expanded JD‐R framework. This gap is particularly notable compared to the extensive research on workload’s impact on job performance [26], which has primarily focused on job demands without considering individual motivational orientations or shift pattern configurations. Therefore, the aim of this quantitative study is to examine whether regulatory focuses, shift patterns, job demands, and job resources predict nurses’ presenteeism and work engagement.

This study addresses the following research questions: (1) How do job demands and resources relate to nurses’ presenteeism and work engagement? (2) How do individual differences in regulatory focus influence these outcomes? (3) Does shift work pattern moderate the relationship between regulatory focus, job characteristics, and outcomes?

## 2. Materials and Methods

### 2.1. Research Design and Sampling

A descriptive, cross‐sectional design was used in this study. The study population consisted of nurses of a tertiary hospital with approximately 2000 beds located in a major metropolitan area in South Korea. Inclusion criteria were (1) registered nurses employed at the study hospital; (2) directly involved in patient care; (3) working rotating shift schedules; (4) assigned to medical/surgical wards, comprehensive nursing care units, intensive care units, or emergency departments; and (5) willing to participate voluntarily. Exclusion criteria were (1) nurses serving as preceptors to avoid potential differences in workload and responsibilities; (2) newly hired nurses in preceptee orientation programs; and (3) nurses in administrative or nonclinical roles. Shift patterns were categorized by Group 1 as 8 h 3‐shift rotation (day, evening, and night shifts), Group 2 as 8 h 2‐shift rotation (day and night shifts or evening and night shifts), and Group 3 as 12 h 2‐shift rotation (rotating between day and night shifts). A quota sampling method was used to ensure sufficient representation of different ward types and shift patterns.

A priori power analysis for multiple regression was conducted using G∗Power 3.1 software [27]. The analysis evaluated the required sample size to detect a medium effect size (*f*2 = 0.15) with a significance level (*α*) at 0.05 and power (1 − *β*) at 0.80, based on 13 predictors in the regression model. The calculated results indicated that a sample size of 131 would achieve the desired power of 0.80.

Considering potential participant dropout and missing data, quota sampling was used to allocate a maximum 13 participants to each sampling cell defined as each unique combination of ward type and shift pattern (e.g., intensive care unit nurses on 8 h 3‐shift rotation, medical ward nurses on 12 h 2‐shift rotation, etc.). Recruitment was conducted through voluntary participation of nurses working at the study hospital during the study period. Permission was obtained from the hospital nursing administration and ward managers. Nurses meeting inclusion criteria were informed about the study through informational flyers posted in nursing stations. Recruitment for each sampling cell was closed when the quota of 13 participants was reached. This study was reported in accordance with Strengthening the Reporting of Observational Studies in Epidemiology (STROBE) guidelines for cross‐sectional studies.

### 2.2. Data Collection Procedures

The data collection was conducted from November 15, 2021, to May 15, 2022, by the principal investigator. Each participant’s data collection followed a two‐stage process: (1) baseline questionnaire completion at enrollment and (2) heart rate monitoring across multiple shifts. The total time required for each participant ranged from 2 weeks to 2 months, depending on their shift rotation schedule and the time needed to capture all required shift types.

This variation in data collection timeline was necessary to capture heart rate measurement across all required shift types for each participant (e.g., day, evening, and night shifts for 8 h 3‐shift rotation nurses). All self‐report measures were completed at a single time point at the beginning of each participant’s enrollment, reflecting their experiences during the past month. Therefore, the variation in timeline affected only the duration needed to complete heart rate monitoring across different shift types, not the assessment of psychological constructs.

At enrollment, participants completed all self‐report questionnaires in a single session. Paper‐based questionnaires were administered in sealed envelopes to ensure confidentiality. These questionnaires assessed demographic characteristics, ward assignment, emotional job demands, job resources, regulatory focus, presenteeism, and work engagement based on experiences during the past month. Following baseline questionnaire completion, physical job demands were measured through heart rate monitoring during an actual work shift. The measured data were collected through an application linked to a smartphone. Only weekday shifts were included, while weekends and public holidays were excluded to ensure consistency in workload conditions.

The data collection procedure for each participant followed these steps. After providing informed consent, participants received the questionnaire packet, a smart band device for heart rate monitoring, and instructions for installing the smartphone application, operating the smart band, and completing the questionnaires. Participants identified upcoming weekday shifts when heart rate monitoring could be conducted. For example, nurses working an 8 h 3‐shift rotation selected one day shift, one evening shift, and one night shift.

On each designated shift, participants wore the smart band from the beginning to the end of their shift. Heart rate data were automatically recorded via the smartphone application. The researcher sent text message reminders on the morning of each scheduled measurement shift. After completing all scheduled heart rate measurements, participants returned the enclosed envelope with completed questionnaires and smart band device to the researcher, and heart rate data were downloaded from the application.

### 2.3. Instruments and Measures

The two aspects of job demand, physical and emotional demands, were measured using heart rate during shift work and self‐report questionnaires, respectively. Job resources were measured by self‐report questionnaires on relationships with colleagues and supervisors, which were social resources. The Questionnaire on the Experience and Evaluation of Work 2.0 (QEEW 2.0) is a comprehensive psychosocial work assessment instrument consisting of 135 items across 42 scales [28]. For this study, three subscales were selected from the QEEW 2.0 based on the JD‐R theoretical framework. The Emotional Demands subscale was used to measure emotional job demands, and the Relationship with Colleagues and Supervisor subscale was used to measure social support. Individual motivational orientation was assessed using the Regulatory Focus Scale (RFS) [29], presenteeism was assessed using the Stanford Presenteeism Scale‐6 (SPS‐6) [30], and work engagement was assessed using the Utrecht Work Engagement Scale‐9 (UWES‐9) [13]. The various response ranges were recoded using basic linear algebra to a 4‐point format across instruments to achieve consistency and facilitate cross‐construct comparisons. This mathematical transformation maintains proportional intervals between response categories and preserves the ordinal properties of the original scales. Internal consistency reliability calculated using recoded responses remained acceptable to good, indicating the recoding did not compromise measurement quality. The formula is following: new score = (original score − minimum value) ∗ (3/[original range]) + 1.

### 2.4. Physical Demands

Physical job demands were assessed objectively using heart rate. The resting heart rate (HR_rest_) was measured once during the initial study enrollment session while the participant was seated and at rest. The work heart rate was measured continuously throughout each designated work shift as exercise heart rate (HR_exercise_). The HR_rest_ is the heart rate per minute measured in a resting state, and the HR_exercise_ is the average heart rate per minute during the shift. The calculation of heart rate reserve (HRR) is given by the following formula (1) [31].

(1) HRR=220−age−HRrest.

Note: HRR = heart rate reserve, HR_rest_ = resting heart rate.

The HR_rest_ is a value that takes into account the subject’s usual fitness based on age, as shown in the equation, and the intensity of the activity can be assessed through the percentage of HRR (%HRR), which is the current heart rate condition relative to the HR_rest_ [32]. The closer the value of the %HRR is to 0, the same as the HR_rest_, and the closer it is to 100, the more intense the physical activity. The formula for the %HRR is as follows (2) [31]. In this study, %HRR was calculated using time T as the peak heart rate per minute during work. Physical job demands were quantified using HR_excersie_ and %HRR.

(2) %HRR=HRT−HRrestHRR×100.

Note: %HRR = percentage of heart rate reserve, HR_*T*_ = heart rate at time *T* = maximum heart rate during the shift.

Heart rate measurement was performed using the Xiomi Miband 5, which is Bluetooth‐connected to a smartphone application to record measurements. The heart rate measurement uses built‐in photoplethysmography, which has a mean absolute percentage error (MAPE) of 6.04%–7.69% and a concordance correlation coefficient (CCC) of 0.76 in previous studies [33]. The smaller the value of MAPE, which measures the prediction accuracy by dividing the difference between the actual value and the predicted value by the actual value, and the closer the value of CCC, which measures the measurement error, is to 1, the higher the confidence.

### 2.5. Emotional Demands

The five items on emotional job demands from the QEEW 2.0 instrument were used (reliability *ρ* = 0.80) [28]. The instrument uses a 4‐point frequency scale (1 = strongly disagree, 4 = strongly agree). Sample items included the following: “My work demands a lot from me emotionally” (general emotional workload), and “In my work, I have contact with difficult customers or patients” (people‐related emotional workload). Cronbach’s α in this study was 0.86.

### 2.6. Job Resource

In QEEW 2.0, social support was examined with six items of relationship with colleagues (reliability *ρ* = 0.81) and six items of relationship with supervisor (reliability *ρ* = 0.87) [28]. The instrument uses a 4‐point frequency scale (1 = strongly disagree, 4 = strongly agree). Sample items included the following: “There have been some unpleasant occurrences between me and my colleagues” (reversed scoring, relationship with colleagues) and “I can count on my supervisor when I come across difficulties in my work” (relationship with supervisor). Cronbach’s *α* in this study was 0.82.

### 2.7. Regulatory Focus

The RFS was used to assess individual motivational orientation, consisting of five questions each for promotion and prevention focus was used (Cronbach’s *α* = 0.79, and 0.67 each, respectively) [34]. Each participant’s dominant regulatory focus was determined by calculating the difference score between promotion focus and prevention focus subscales. A positive difference score indicates a promotion‐dominant focus, while negative scores indicate a prevention‐dominant focus. The original instrument uses a 5‐point Likert scale (1 = strongly disagree, 5 = strongly agree), and responses were recoded to a 4‐point scale. Sample items included the following: “I am confident that I can achieve my hopes and aspirations through work” (promotion focus), and “I am concerned that I am falling short of work responsibilities and duties” (prevention focus). In this study, Cronbach’s α for the promotion focus and prevention focus were 0.74 and 0.64, respectively.

### 2.8. Presenteeism

It was measured using the SPS‐6, which consists of six questions (Cronbach’s *α* = 0.67) [30]. The SPS‐6 asks to what extent the health problem that affects your daily life the most affects your work performance. The SPS‐6 uses a 5‐point Likert scale (1 = strongly disagree, 5 = strongly agree), and responses were recoded to a 4‐point scale. Sample items included the following: “Despite having my health problem, I was able to finish hard tasks in my work” (reversed scoring), and “Because of my health problem, the stresses of my job were much harder to handle”. Cronbach’s α in this study was 0.70.

### 2.9. Work Engagement

The UWES‐9 is a shortened version of the UWES and consists of nine items, three each for the three subitems of vigor, dedication, and absorption that constitute work engagement (Cronbach’s *α* = 0.93) [13]. The instrument uses a 7‐point frequency scale (0 = never, 6 = always) and responses were recoded to a 4‐point scale. Sample items included the following: “At my work, I feel bursting with energy”, “I am proud of the work that I do”, and “I feel happy when I am working intensely”. Cronbach’s α in this study was 0.69.

### 2.10. Analysis

Completed questionnaires underwent review for completeness and consistency. Cases with missing data on key study variables were excluded from analysis. Descriptive statistics were calculated for all demographic characteristics and study variables.

Prior to hypothesis testing, the dataset was assessed for statistical assumptions. Internal consistency reliability of all instruments was evaluated using Cronbach’s alpha coefficient. Normality of continuous variables was assessed using the Shapiro–Wilk test and visual inspection of Quantile–Quantile (Q‐Q) plots. Multicollinearity among independent variables was examined using variance inflation factors (VIF).

Presenteeism and work engagement were examined as dependent variables. Job demands, including physical (HR_exercise_ and %HRR) and emotional demands, job resources such as relationship with colleagues and supervisor, three types of shift patterns, and dominant regulatory focus were independent variables. Control variables were clinical experience and unit type. Separated hierarchical multiple regression analysis was conducted to examine the incremental contribution of different variable sets in predicting presenteeism and work engagement. This approach allows assessment of whether additional variables explain significant variance in the outcomes beyond what is explained by variables entered in earlier steps.

For each model, regression coefficients, standardized regression coefficients, standard errors, and *p* values were calculated. Model fit was assessed using adjusted *R* ^2^, which indicates the proportion of variance in the dependent variable explained by the independent variables. The incremental change in *R* ^2^ between successive models was tested for statistical significance using ANOVA F‐tests. All statistical analyses were conducted using R version 4.3.3 [35]. Results were considered significant at a 95% confidence interval, *p* < 0.05.

### 2.11. Ethical Considerations

The study received institutional review board approval from Samsung Medical Center (IRB No.2021‐05‐119‐001). The cover letter served as the informed consent document, emphasizing the voluntary nature of participation and the right to withdraw at any time without consequences. Participants were provided with information about the study purpose, procedures, potential risks and benefits, and contact information for questions or concerns. The researcher ensured minimal disruption of routine clinical work and additional workplace burden for participants. Once a participant completed data collection and returned the smart band device, their questionnaire data were immediately de‐identified by removing all personal identifiers and assigning a random numeric code. During the data collection period, a master list linking participants to their assigned study data were maintained in a password‐protected, encrypted file accessible only to the principal investigator. This master list was permanently destroyed immediately after each participant’s data were de‐identified. All data were kept secure and confidential and was accessed by the researcher only. After the retention period in accordance with institutional policy, all study data will be permanently destroyed. Paper consent forms were stored separately from study data in a locked filing cabinet in the principal investigator’s office.

## 3. Results

### 3.1. Demographic Characteristics and Descriptive Statistics

A total of 185 participants were recruited from 5 ward types and 3 shift patterns, and after excluding those who dropped out due to shift changes and those who returned questionnaires with nonresponses, a final sample of 176 participants was analyzed (95% response rate). The majority of the study participants were female (94.9%), with 105 (59.7%) in their 20s. The average clinical experience of the study participants was 73.2 ± 40.9 months, or approximately 6 years. The study included 60 nurses working 8 h 3‐shift rotation (Group 1), 60 nurses working an 8 h 2‐shift rotation (Group 2), and 56 nurses working two 12 h 2‐shift rotation (Group 3), with 37 nurses from internal, 35 from surgery, 36 from comprehensive nursing care units, 35 from the emergency room, and 33 from the intensive care unit (Table 1). The units of participants varied in size and staffing patterns. Total nursing staff per unit ranged from 17 to 97 nurses. During individual shifts, nurse‐to‐patient ratios ranged from 1:2 in intensive care settings to 1:12 in general medical/surgical wards, depending on the unit type and acuity.

**TABLE 1:** Demographic characteristics of nurses (*n* = 176).

Characteristics	M ± SD or *n* (%)
Age (years)	29.3 ± 3.54
Duration of working in nursing (months)	73.2 ± 40.9
Duration of working in current ward (months)	41.5 ± 26.4
Gender	
Female	167 (94.9)
Male	9 (5.1)
Marital status	
Single	143 (81.3)
Married	33 (18.7)
Education	
Bachelor’s degree	173 (98.3)
Master’s degree	3 (1.7)
Unit type	
Medical ward	37 (21.0)
Surgical ward	35 (19.9)
Comprehensive nursing care unit	36 (20.5)
Emergency room	35 (19.9)
Intensive care unit	33 (18.7)
Shift pattern	
8 h 3‐shift rotation (Group 1)	60 (34.1)
8 h 2‐shift rotation (Group 2)	60 (34.1)
12 h 2‐shift rotation (Group 3)	56 (31.8)

Descriptive statistics of nurses′ physical and emotional job demands, presenteeism, and work engagement are shown in Table 2. On a 4‐point scale, nurses reported high levels of job resources, with mean scores of 3.2 (SD 0.5) for relationship with colleagues and 3.1 (SD 0.6) for relationship with supervisor. For regulatory focus, the mean dominant regulatory focus score was 1.6 (SD 3.1), with positive values indicating a prevention‐dominant orientation. When categorized based on their dominant regulatory focus score, 63.1% of nurses (*n* = 111) were classified as prevention dominant (score > 0), while 36.9% (*n* = 65) were classified as promotion dominant (score < 0). Among the prevention‐dominant subgroup, the mean prevention subscale score was 2.5 (SD 1.4).

**TABLE 2:** Mean of study variables (*n* = 176).

	M ± SD (min–max)
Physical job demand	
HR_exercise_ (rate per min)	89.6 ± 6.6 (77–103)
%HRR (%)	49.6 ± 9.7 (29–79)
Emotional demand	2.7 ± 0.8
Job resource	
Relationship with colleagues	3.2 ± 0.5
Relationship with supervisor	3.1 ± 0.6
Dominant regulatory focus	1.6 ± 3.1 (−5–9)
Presenteeism	2.3 ± 0.5
Work engagement	2.2 ± 0.5

*Note:* HR_exercise_: exercise heart rate.

Abbreviation: %HRR, percentage of heart rate reserve.

### 3.2. Hierarchical Regression

The effects of job demands, job resources, shift patterns, and dominant regulatory focus on presenteeism and work engagement are presented in Table 3. Hierarchical multiple regression analyses were conducted to examine factors predicting presenteeism and work engagement. Three sequential models were tested: Model 1 included control variables, job demands, and job resources; Model 2 added a shift pattern; and Model 3 added a dominant regulatory focus. The control variables were the subjects’ experience and the work environment factor, ward. The VIF values of the included variables were all less than 10 (1.15∼7.64). The dominant regulatory focus is the value obtained by subtracting the promotion focus from the prevention focus, and a larger positive value indicates a stronger tendency toward prevention‐dominant focus, and a larger negative value indicates a stronger tendency toward promotion‐dominant focus.

**TABLE 3:** The effects of job demands, resources, shift types, and dominant regulatory focus on presenteeism and work engagement: hierarchical regression (*n* = 176).

Independent variables	Dependent variables																	
	Presenteeism									Work engagement								
	Model 1			Model 2			Model 3			Model 1			Model 2			Model 3		
	B	SE	*β*	B	SE	*β*	B	SE	*β*	B	SE	*β*	B	SE	*β*	B	SE	*β*
Years as a nurse (vs > 5)	0.01	0.08	0.01	0.00	0.07	0.00	0.03	0.07	0.02	0.00	0.07	0.00	0.00	0.06	0.00	−0.05	0.05	−0.05
Unit (vs medical ward)																		
Surgical ward	0.24	0.14	0.17	0.18	0.14	0.13	0.13	0.13	0.09	0.05	0.12	0.04	−0.01	0.12	−0.01	0.09	0.10	0.08
Comprehensive nursing care unit	−0.06	0.12	−0.05	−0.08	0.11	−0.06	−0.07	0.11	−0.05	−0.03	0.10	−0.02	−0.04	0.10	−0.04	−0.07	0.08	−0.06
Emergency room	0.03	0.12	0.02	0.05	0.11	0.03	0.02	0.11	0.01	−0.22	0.10	−0.19	−0.21	0.10	−0.18	−0.15	0.08	−0.13
Intensive care unit	0.11	0.12	0.08	0.08	0.12	0.06	0.07	0.12	0.05	0.15	0.11	0.13	0.13	0.10	0.11	0.15	0.08	0.13
HR_exercise_	0.03^∗^	0.01	0.32	0.04^∗^	0.01	0.34	0.03^∗^	0.01	0.28	−0.01	0.01	−0.08	−0.01	0.01	−0.07	0.01	0.01	0.08
%HRR	0.02^∗^	0.00	0.32	0.02^∗^	0.00	0.28	0.02^∗^	0.00	0.28	0.00	0.00	0.08	0.00	0.00	0.04	0.00	0.00	0.05
Emotional demand	0.13^∗^	0.06	0.18	0.09	0.06	0.13	0.07	0.06	0.09	0.04	0.06	0.07	0.01	0.05	0.01	0.06	0.04	0.10
Relationship with colleagues	0.01	0.09	0.13	−0.03	0.09	−0.23	0.03	0.09	0.02	0.14	0.08	0.15	0.09	0.08	0.10	−0.01	0.07	−0.01
Relationship with supervisor	−0.13	0.09	−0.13	−0.13	0.09	−0.13	−0.03	0.09	−0.03	0.25^∗^	0.08	0.30	0.25^∗^	0.08	0.23	0.04^∗^	0.07	0.04
Shift pattern (vs Group 3)																		
Group 1				−0.31^∗^	0.11	−0.21	−0.28^∗^	0.11	−0.20				−0.32^∗^	0.10	−0.26	−0.38^∗^	0.08	−0.31
Group 2				−0.25^∗^	0.08	−0.23	−0.26^∗^	0.08	−0.24				−0.22^∗^	0.07	−0.24	−0.20^∗^	0.06	−0.22
Dominant regulatory focus							0.04^∗^	0.01	0.25							−0.09^∗^	0.01	−0.60
*F*	6.436^∗^			6.653^∗^			7.359^∗^			4.836^∗^			5.517^∗^			14.560^∗^		
*R* ^2^	0.281			0.329			0.371			0.227			0.289			0.539		
Adjusted *R* ^2^	0.237			0.279			0.321			0.180			0.237			0.502		
Δ*R* ^2^				0.042^∗^			0.041^∗^						0.057^∗^			0.265^∗^		

*Note:* Group 1: 8 h 3‐shift rotation; Group 2: 8 h 2‐shift rotation; Group 3 (ref): 12 h 2‐shift rotation. HR_exercise_: exercise heart rate.

Abbreviation: %HRR, percentage of heart rate reserve.

^∗^ *p* < 0.05.

Model 1 tested the basic JD‐R model propositions by examining the effects of job demands and job resources on presenteeism and work engagement, controlling for clinical experience and unit type. Physical job demands significantly predicted higher presenteeism, with both HR_exercise_ (*β* = 0.32, *p* < 0.05) and %HRR (*β* = 0.32, *p* < 0.05) showing positive associations. Emotional demands also significantly predicted higher presenteeism (*β* = 0.18, *p* < 0.05). However, neither relationship with colleagues nor with supervisor significantly predicted presenteeism (*p* > 0.05). Model 1 explained 23.7% of variance in presenteeism (adjusted *R* ^2^ = 0.2370, *F* = 6.436, *p* < 0.05). While a relationship with a supervisor significantly predicted higher work engagement (*β* = 0.30, *p* < 0.05). Physical job demands, emotional demands, and relationship with colleagues did not significantly predict work engagement in this model (*p* > 0.05). Model 1 explained 18.0% of the variance in work engagement (adjusted *R* ^2^ = 0.1798, *F* = 4.836, *p* < 0.05).

Model 2 tested whether shift pattern explained additional variance in presenteeism and work engagement beyond the effects of job demands and resources. The addition of shift pattern significantly improved model fit (Δ*R* ^2^ = 0.0424, *p* < 0.05) for presenteeism. Compared to nurses working 12 h 2‐shift rotation, nurses working 8 h 3‐shift rotation (*β* = −0.21, *p* < 0.05) and 8 h 2‐shift rotation (*β* = −0.23, *p* < 0.05) reported significantly lower presenteeism. The pattern of job demands and resources effects remained similar to Model 1. Model 2 explained 28.0% of variance in presenteeism. The addition of a shift work pattern also significantly improved model fit for work engagement (Δ*R* ^2^ = 0.0567, *p* < 0.05). Compared to nurses working 12 h 2‐shift rotation, nurses working 8‐h 3‐shift rotation (*β* = −0.26, *p* < 0.05) and 8 h 2‐shift rotation (*β* = −0.24, *p* < 0.05) reported significantly lower work engagement. A relationship with a supervisor remained a significant predictor. Model 2 explained 23.7% of the variance in work engagement.

Finally, Model 3 tested whether dominant regulatory focus explained additional variance beyond job characteristics and shift pattern. The addition of regulatory focus significantly improved model fit for presenteeism (Δ*R* ^2^ = 0.0414, *p* < 0.05). A prevention‐dominant focus significantly predicted higher presenteeism (*β* = 0.25, *p* < 0.05), meaning that nurses with a stronger prevention focus experienced more presenteeism. The effects of physical job demands, emotional demands, and shift work patterns from Model 2 remained statistically significant. Model 3 explained 32.1% of the variance in presenteeism (adjusted *R* ^2^ = 0.3208, *F* = 7.359, *p* < 0.05). The addition of regulatory focus substantially improved model fit for work engagement (Δ*R* ^2^ = 0.2654, *p* < 0.05). A prevention‐dominant focus strongly predicted lower work engagement (*β* = −0.60, *p* < 0.05), indicating that nurses with a stronger prevention focus experienced significantly lower engagement. This was the strongest predictor in the model. The effects of relationship with supervisor and shift pattern from Model 2 remained significant. Model 3 explained 50.2% of the variance in work engagement (adjusted *R* ^2^ = 0.5019, *F* = 14.560, *p* < 0.05).

For presenteeism, the addition of shift pattern (Model 2) and regulatory focus (Model 3) each contributed statistically significant but modest improvements in explanatory power (4.2% and 4.1%, respectively). The final model (Model 3) explained 32.1% of variance in presenteeism, with physical job demands, shift pattern, and regulatory focus emerging as statistically significant predictors. For work engagement, regulatory focus provided a substantial 26.5%‐point increase in explained variance, demonstrating the critical importance of individual motivational orientation. The final model explained 50.2% of variance in work engagement, with regulatory focus emerging as the strongest predictor, followed by relationship with supervisor and shift pattern.

## 4. Discussion

This study expanded the JD‐R model by integrating shift patterns and individual regulatory focus to examine predictors of presenteeism and work engagement among hospital nurses. Results demonstrated that physical job demands, shift patterns, and prevention‐dominant focus significantly predicted presenteeism, while the relationship with supervisor, shift patterns, and prevention‐dominant focus predicted work engagement. Dominant regulatory focus emerged as a particularly strong predictor of work engagement, explaining the additional variance beyond job characteristics and shift patterns. These findings highlight the importance of considering both shift patterns and motivational orientation when addressing nurse well‐being and performance.

The findings of this study can be interpreted within the dual‐process framework of the JD‐R model. The health impairment process, in which job demands deplete workers’ energy and lead to negative outcomes, was evident in the significant positive relationship between physical job demands and presenteeism. Nurses experiencing higher physical demands, as measured by heart rate indicators during shift work, were more likely to work while ill, supporting the notion that high demands create strain that manifests as presenteeism. Conversely, the motivational process, in which job resources foster engagement and positive outcomes, was demonstrated through the significant relationship between supervisor support and work engagement. This aligns with the JD‐R model’s proposition that resources activate motivational processes leading to positive work outcomes.

Importantly, this study extended the JD‐R model by incorporating both organizational factors and personal resources, as called for in recent theoretical developments [15, 25]. Shift patterns functioned as an organizational‐level factor that influenced both negative outcomes and positive outcomes, suggesting that shift work acts as a contextual demand that shapes how nurses experience other job characteristics. The dominant regulatory focus, representing a personal resource, emerged as the strongest predictor of work engagement, explaining additional variance beyond organizational job demands, resources, and shift patterns. This finding demonstrates that personal motivational orientations play a critical role in the motivational process of the extended JD‐R model, potentially even exceeding the influence of traditional job characteristics in predicting positive outcomes like engagement.

The results of this study showed that as physical job demands increased, nurses’ presenteeism also increased. However, in the final model, Model 3, the effect of emotional job demands on presenteeism was not statistically significant. The effect of job resources on presenteeism was also not statistically significant, and only the relationship with the supervisor had a significant effect on work engagement. However, by adding shift patterns and dominant regulatory focus variables to the existing model, the explanatory power of each model was statistically significantly improved.

In detail, the %HRR during the nurses’ work hours was moderate intensity (40 ≤ %HRR < 60) [36]. This is higher than in studies of manual workers, and previous studies have shown that %HRR increases more when there are physical job demands [37, 38]. The higher prevention‐dominant focus than promotion‐dominant focus differs from previous studies that have found a promotion focus to be higher than a prevention focus in other occupations [39]. This reflects the nature of nursing, where error prevention is paramount in patient care. This is because tertiary hospitals, where patients are admitted and treated primarily in the acute phase of their illness, have a higher level of patient acuity, and nurse and patient safety incidents can have a negative impact on patient outcomes.

However, emotional job demands and job resources do not have a significant effect on presenteeism. There may be cultural differences in emotional job demands and social support. In fact, there is a large difference in the rate of sick workers coming to work and sick days off between OECD countries and Korean wage workers [40]. This suggests there may be large differences in workers’ perceptions of presenteeism and absenteeism, as well as in corporate culture. In addition, the finding that a relationship with a supervisor significantly predicted work engagement differs from previous research [41]. However, other studies have found a relationship with a supervisor to be a critical predictor of engagement, particularly in contexts where supervisors directly influence work assignments and professional development opportunities [42]. These mixed findings suggest that the relative importance of colleague versus supervisor relationships may vary across different healthcare settings, national contexts, or organizational cultures. Therefore, additional research is needed to clarify the contextual factors that determine when a relationship of a supervisor versus colleagues has greater influence on nurses’ work engagement.

Meanwhile, the presenteeism of nurses working 12 h shifts was found to be higher than that of nurses working 8 h shifts. This result was similar to a previous study that found that presenteeism increased as working hours increased [43]. On the other hand, nurses working 12 h shifts also reported higher work engagement than nurses working 8 h shifts. This is consistent with previous research that suggests that 12 h shifts have a positive effect on work organization [44], which suggests that nurses who choose to work 12 h shifts are more likely to be engaged in their jobs. However, working more than 12 h a day has negative effects on nurses and patients [7], so it needs to be managed. This should be supported by policies that ensure minimum rest periods after shifts, limit consecutive shifts exceeding 4 days, ensure consecutive days off following consecutive shifts, and allow nurses to choose their preferred shift patterns.

The 8 h 2‐shift rotation pattern examined in this study warrants additional discussion, while 8 h shifts are standard in many countries, the specific configuration of 8 h 2‐shift rotation is less commonly studied in the nursing literature compared to the traditional 8 h 3‐shift rotation. Most shift work research has focused on comparing shift workers to nonshift workers or examining differences in shift length, rather than comparing different rotation patterns among nurses working the same shift length. This limited existing research makes it difficult to contextualize findings regarding the 8 h 2‐shift rotation pattern. Because presenteeism tends to be higher in shift workers compared to fixed‐schedule workers [45], further research is needed to determine whether reducing the number of shift rotations from 3 to 2 can alleviate the negative effects of shift work or whether this pattern has unique implications for nurses and patient outcomes.

The findings regarding regulatory focus indicate that prevention‐dominant focus significantly predicted both higher presenteeism and lower work engagement, with particularly strong effects on engagement. According to regulatory focus theory [17], prevention‐focused individuals are motivated by duties, responsibilities, and loss avoidance through vigilant strategies, while promotion‐focused individuals pursue ideals and gains through eager strategies. In nursing, a prevention‐dominant focus may contribute to presenteeism because nurses oriented toward fulfilling obligations may feel compelled to work even when ill, viewing absence as failing to meet responsibilities. The vigilant cognitive style of prevention focus may also create chronic psychological strain as nurses constantly monitor for errors, potentially depleting resources and increasing vulnerability to presenteeism.

The strong negative relationship between prevention‐dominant focus and work engagement aligns with theoretical predictions. Work engagement emerges from approach‐oriented, growth‐focused motivation characteristic of promotion focus [13, 17]. Prevention‐focused nurses, oriented toward maintaining security and fulfilling duties, may experience work as an obligation rather than an energizing pursuit of ideals, leading to lower engagement. This is supported by previous research linking prevention focus to burnout and promotion focus to engagement [46].

The substantial variance explained by regulatory focus suggests individual motivational orientation may be as important as organizational characteristics. This implies interventions should address both organizational factors and individual motivation. Organizations might cultivate promotion focus by emphasizing growth opportunities and professional development alongside necessary safety vigilance. Future research should explore interventions that balance both regulatory foci to maintain safety while fostering engagement.

This study advances the literature in three important ways. First, it integrates regulatory focus theory with the JD‐R model to examine nurses’ well‐being and performance. The finding that dominant regulatory focus explained an additional variance in work engagement demonstrates that individual motivational orientations are at least as important as organizational job characteristics in predicting nurse outcomes.

Second, this study provides novel evidence on shift rotation patterns by comparing different rotation configurations with the same shift length, which previous research has largely overlooked. The finding that rotation pattern matters beyond shift length has practical implications for optimizing nursing schedules.

Third, by simultaneously examining both presenteeism and work engagement with differential patterns of predictors, this study demonstrates that interventions targeting negative outcomes may need to differ from those enhancing positive outcomes.

These findings have practical implications for organizations seeking effective strategies to address presenteeism and enhance work engagement among nurses. The time‐sensitive nature of nursing work means that tasks cannot be delayed. Without a flexible workforce, it becomes difficult to be absent from work even when sick, leading to presenteeism. Previous research has shown that these institutional and support barriers limit individual motivation and engagement [47]. Therefore, flexible and appropriate nursing staffing and optimizing shift schedules must be combined with organizational‐level policy support and resource allocation. Additionally, nurse manager support and positive motivation are essential for preventing presenteeism, enhancing work engagement, and helping nurses explore their aptitudes and advance their careers. Based on these findings, future studies should explore which interventions effectively strengthen or mitigate dominant regulatory focus and whether interventions targeting specific regulatory focuses actually reduce presenteeism and increase work engagement.

### 4.1. Limitation

First, the cross‐sectional design does not allow inferring causality. Second, this study was conducted among nurses in a single tertiary hospital. Therefore, despite adequate power analysis, external validity remains limited. Third, the sample was predominantly female, which limits the generalizability of findings to male nurses. Future research should employ multicenter longitudinal panel studies using stratified sampling and a quasi‐experimental design to establish causal relationships between variables.

## 5. Conclusion

This study identified how job demands and resources including shift type and dominant regulatory focus influence nurse presenteeism and work engagement. The results indicate that physical job demands, shift patterns, and prevention‐dominant focus influence presenteeism, while promotion‐dominant focus affects work engagement. Therefore, organizational strategies that manage physical job demands, support flexible shift work schedules, and promote promotion focus are needed to reduce presenteeism and improve work engagement.

## Funding

This research received no specific grant from any funding agency in the public, commercial, or not‐for‐profit sectors.

## Conflicts of Interest

The author declares no conflicts of interest.

## Supporting Information

Supporting File 1 contains the STROBE checklist for the reporting of cross‐sectional studies.

## Supporting information


**Supporting Information** Additional supporting information can be found online in the Supporting Information section.

## Data Availability

The data that support the findings of this study are available on request from the corresponding author. The data are not publicly available due to privacy or ethical restrictions.
